# Identification of KIT and BRAF mutations in thyroid tissue using next-generation sequencing in an Ecuadorian patient: A case report

**DOI:** 10.3389/fonc.2022.1101530

**Published:** 2023-01-17

**Authors:** Santiago Cadena-Ullauri, Elius Paz-Cruz, Rafael Tamayo-Trujillo, Patricia Guevara-Ramírez, Viviana Ruiz-Pozo, Paola Solis-Pazmino, Cristhian Garcia, Richard Godoy, Eddy Lincango-Naranjo, Ana Karina Zambrano

**Affiliations:** ^1^ Centro de Investigación Genética y Genómica, Facultad de Ciencias de la Salud Eugenio Espejo, Universidad UTE, Quito, Ecuador; ^2^ Surgery Group of Los Angeles, Department of Colorectal Surgery, Los Angeles, CA, United States; ^3^ Instituto de la Tiroides y Enfermedades de Cabeza y Cuello (ITECC), Department of Head and Neck Surgery, Quito, Ecuador; ^4^ Department of Teaching and Research, Hospital Vozandes, Quito, Ecuador; ^5^ CaTaLiNA Research Initiative (Cáncer de tiroides en Latinoamérica), Quito, Ecuador

**Keywords:** genomics, thyroid, cancer, mestizo, case report

## Abstract

**Background:**

The incidence of thyroid cancer has increased worldwide. Ecuador presents the highest incidence among Latin American countries and the second around the world. Genetic alteration is the driving force for thyroid tumorigenesis and progression. The change from valine (V) to glutamic acid (E) at codon 600 of the BRAF gene (BRAF^Val600Glu^) is the most commonly reported mutation in thyroid cancer. Moreover, the BRAF mutation is not the only mutation that has been correlated with TC. For instance, mutations and overexpression of the KIT gene has been associated with different types of cancer, including lung and colon cancer, and neuroblastoma.

**Case presentation:**

A woman in her early fifties, self-identified as mestizo, from Otavalo, Imbabura-Ecuador had no systemic diseases and denied allergies, but she had a family history of a benign thyroid nodule. Physical examination revealed a thyroid gland enlargement. The fine-needle aspiration biopsy indicated papillary thyroid cancer. The patient underwent a successful total thyroidectomy with an excellent recovery and no additional treatments after surgery. Using Next-Generation sequencing a heterozygous mutation in the BRAF gene, causing an amino acid change Val600Glu was identified. Similarly, in the KIT gene, a heterozygous mutation resulting in an amino acid change Leu678Phe was detected. Moreover, an ancestry analysis was performed, and the results showed 3.1% African, 20.9% European, and 76% Native American ancestry.

**Conclusions:**

This report represents the genetic characteristics of papillary thyroid cancer in an Ecuadorian woman with a mainly Native American ethnic component. Further studies of pathological variants are needed to determine if the combined demographic and molecular profiles are useful to develop targeted treatments focused on the Ecuadorian population.

## Background

The incidence of thyroid cancer (TC) has increased worldwide (1). In the United States, thyroid cancer incidence has tripled over the last three decades from 5.5 to 14.0 per 100,000 people in 2019. TC ranks as the fifth most common cancer in Ecuadorian women (2, 3).

TC is classified into differentiated TC (DTC), poorly differentiated TC (PDTC), and anaplastic TC (ATC) (4). The most common type is differentiated TC (DTC) including papillary thyroid cancer (PTC), and particularly thyroid cancers of 1 cm or less in size, called papillary thyroid microcarcinoma (5). DTC, including PTC, is relatively indolent and highly curable; however, a significant recurrence rate, about 20% at 10 years and 30% at 30 years after initial treatment, is seen.

Genetic alteration is the driving force for thyroid tumorigenesis and progression. The main metabolic pathways associated with TC oncogenesis are the mitogen-activating protein kinase (MAPK) signaling pathway and the Phosphatidylinositol-3-kinase (PI3K)-AKT pathway (4). Mutations in protein components of these metabolic pathways lead to the translocation of transcription factors upregulating gene transcription and promoting oncogenesis 4). The change from valine (V) to glutamic acid (E) at codon 600 of the BRAF gene (BRAF^Val600Glu^) is the most commonly reported mutation in PTC (4, 6). The BRAF mutation leads to the activation of the BRAF kinase in the MAPK pathway; this is considered the initial event in the oncogenesis and progression of PTC (4). An association between the BRAF^Val600Glu^ variant presence and worse prognosis, extrathyroidal extension, and lymph node metastasis has also been established (6, 7). Moreover, the BRAF mutation is not the only mutation that has been correlated with TC. For instance, mutations and overexpression of the KIT gene has been associated with different types of cancer, including lung cancer, neuroblastoma, and colon (8–10). Hence, it is important to identify mutations that along with the BRAF^Val600Glu^ mutation are driving the tumorigenesis.

The present case report describes a woman in her early fifties who underwent total thyroidectomy with malignant histopathologic features and the presence of a BRAF^Val600Glu^ and a KIT^Leu678Phe^ variants.

## Case presentation

The present case report describes a woman in her early fifties, self-identified as mestizo, from Otavalo, Imbabura-Ecuador. In her familial history, the mother and father did not report any type of cancer, and only one of her brothers, among six, presented thyroid nodules; however, they were categorized as benign. The patient attended to the physician due to a lump in the neck that had been gradually enlarging for about 2 years. She had no symptoms of dysphagia and denied odynophagia or shortness of breath. The patient had no systemic diseases and denied allergies.

Physical examination revealed a thyroid gland enlargement. The ultrasound confirmed the thyroid growth (29 x 17 x 22 mm) and showed a solid nodule located in the left lobule with irregular borders, microcalcifications, highly vascularized. The fine-needle aspiration biopsy indicated a Bethesda VI consistent with papillary thyroid cancer. Based on the results, a total thyroidectomy was chosen as the best option (11). The subject’s blood tests were normal; therefore, the patient could undergo surgery. The procedure was successful with an excellent recovery. Posterior medical checkups did not show any complication.

**Figure 1:**
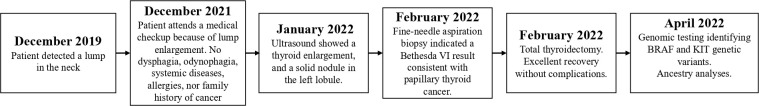
Subject’s relevant episodes of care. The disease-associated episodes of care are presented in the figure.

Moreover, an ancestral composition analysis was performed, and the results showed 3.1% African, 20.9% European, and 76% Native American. A timeline of the relevant episodes of care is depicted in **Figure 1**.

### Genomic analyses

**Figure 2:**
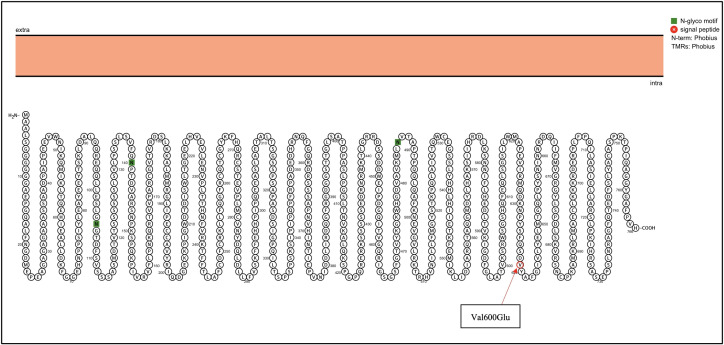
BRAF genetic mutation identified in the patient by NGS. Proteins were visualized with Protter (12). The red circle highlights the identified Val600Glu mutation in the BRAF gene.

**Figure 3:**
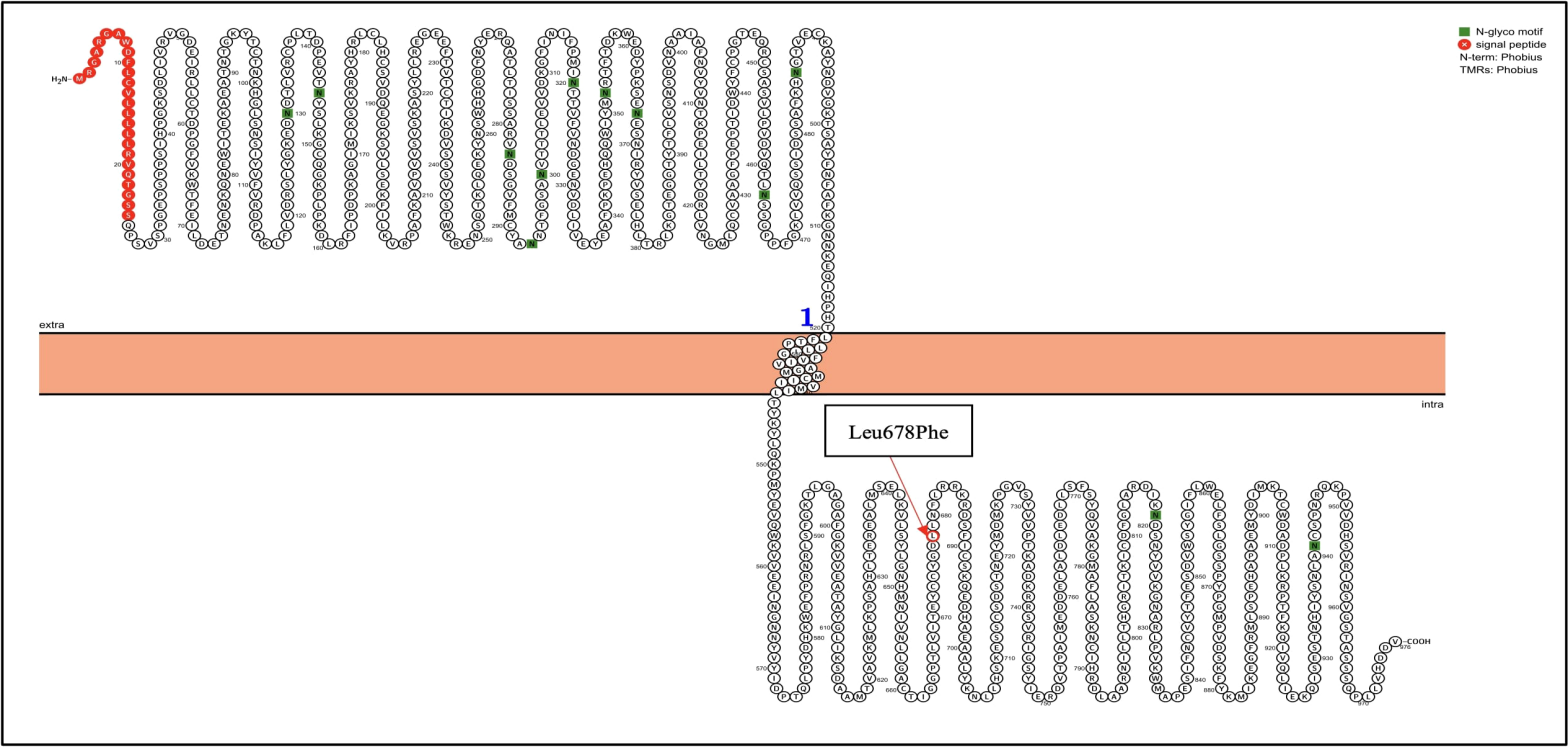
KIT genetic mutation identified in the patient by NGS. Proteins were visualized with Protter (12). The red circle highlights the identified Leu678Phe mutation in the KIT gene.

Next-generation sequencing was performed using the TruSight Tumor 15 kit from Illumina ^®^; this panel includes 15 genes commonly mutated in solid tumors. Among the mutated genes, two had an in silico pathogenic status: BRAF and KIT, as described in the **Table 1**. The BRAF gene suffered a heterozygous mutation, a change from adenine to thymine at position 1799; this caused an amino acid change from Valine to Glutamic acid at position 600 (**Figure 2**), confirmed with Sanger Sequencing. Similarly, in the KIT gene, a heterozygous mutation led to a thymine change to cytosine at position 2032, which resulted in an amino acid change from Leucine to Phenylalanine at position 678 (**Figure 3**).

**Table 1:** Genetic variants identified using next generation sequencing.

Gene	Chromosome	HGVS DNA Reference	HGVS Protein Reference	Predicted Effect	dbSNP/dbVar ID	Genotype
BRAF	7	NM_004333.4:c.1799T>A	NM_004333.4:p.(Val600Glu)	Missense variant	rs113488022	Heterozygous
KIT	4	NM_000222.2:c.2032C>T	NM_000222.2:p.(Leu678Phe)	Missense variant	N/A	Heterozygous

## Methods

### Sample collection and DNA extraction

After the total thyroidectomy procedure, a tissue sample (21.3 mg) was sent to the Centro de Investigación Genética y Genómica for the genomic analyses. The patient’s informed consent was signed before the surgery. The DNA extraction was performed using the PureLink Genomic DNA Mini Kit, according to the manufacturer’s instructions (Invitrogen, USA), and was quantified using spectrophotometry.

### PCR amplification and genotyping for ancestral composition analysis

A multiplex reaction was performed for the amplification of 46 ancestry-informative markers (AIMs)-InDels. The amplification procedure was based on the protocol by Pereira et al. with a 10ul final reaction volume, using a Qiagen Multiplex PCR Master Mix (Qiagen), 46 (AIMs)-InDels primers and genomic DNA (13). Positive DNA control 2800M was included in the reaction along with the patient sample. The PCR amplification program was 95°C for 15 min; 30 cycles at 94°C for 30 sec, 60°C for 90 sec, and 72°C for 45 sec; and a final extension at 72°C for 60 min (13). A 3500 Genetic Analyzer (Applied Biosystems) was used for fragment separation and detection. The results were collected in Data Collection v4.0 and Gene Mapper v5 (Applied Biosystems)

### Statistical analyses

The statistical analyses were performed according to Zambrano, et al. (14) using the software STRUCTURE v2.3.4. The ancestry inferences compared the subject and each reference population from HGDP-CEPH (Africans, Europeans, and Native Americans) subset H952. The run consisted of a burn-in length of 10 000, and 10 000 Markov Chain Monte Carlo (MCMC) interactions.

### Next-generation sequencing

The TruSight Tumor 15 kit from Illumina ^®^ was used for sequencing on a MiSeq platform, according to the manufacturer’s instructions (Illumina, USA). Data analyses, including variant calling, were performed on the TruSight Tumor 15 v2.0.1 for mapping, Pisces 5.1.7.52 for variant caller, Sift and PolyPhen for *in silico* prediction and COSMIC and ClinVar for clinical association.

## Discussion and conclusions

Thyroid cancer (TC) is a malignant tumor with the most rapid increase in the incidence rate in the last three decades (2, 15–20). The incidental finding is a worldwide phenomenon. However, few studies have reported that the prevalence of large and aggressive tumors is also increasing (21–23), suggesting that factors other than overdiagnosis might be affecting the increase of PTC incidence (24–28). Another factor that may be associated with a higher incidence of PTC is the use of radioactive drugs in hyperthyroid patients (29).

Several pieces of research suggest that thyroid cancer incidence is different among populations. In the USA, non-Hispanic Caucasians are less affected than the Hispanic and African-American populations (20, 30). Population-specific factors, such as American Indian ancestry, which influences other cancer patterns, may increase the risk of PTC in the Ecuadorian population (31–33). Moreover, Salazar-Vega et al. (2019) found that the mestizo population had a higher incidence of thyroid cancer in Ecuador (17). In the same study, they found that most of the thyroid cancer patients came from the highlands (2358masl), in comparison of those from the coast (93masl) and Amazon (731masl) regions (17); this is similar to what was found by Zeng, R. et al. (34) where high altitude was correlated with a higher thyroid cancer incidence (34). The subject comes from Otavalo a region located at 2532masl; hence the high altitude could be a risk factor for the presence of her thyroid cancer.

Furthermore, diet has been associated with an increased thyroid cancer predisposition (35). In Ecuador, grains are the most consumed food, especially in the highlands, and starch has been associated with an increased thyroid cancer predisposition (35); therefore, the Ecuadorian diet could also have a role in the increased thyroid cancer incidence.

In Ecuador, genetic studies are scarce. Solis et al. (21). found a BRAF^Val600Glu^ mutation in 130 of 169 (76,9%) PTC patients from the northern Ecuadorian Andes treated at the Hospital Eugenio Espejo in Quito, Ecuador (21), similar to other populations around the world (22, 23, 32, 36–38). The BRAF^Val600Glu^ mutation constitutively activates the MAPK pathway, promoting cell survival, proliferation, and growth, thus tumorigenesis (39, 40). The **Supplementary Figure 1** represents the difference between the wild-type and mutant residues. There is a significant difference in size, hydrophobicity, and charge. Studies *in vitro* have identified a 500-fold greater activity of BRAF^Val600Glu^ in comparison with the wild type (40). The BRAF^Val600Glu^ mutation has been widely described, and nowadays, specific treatments have been designed for this mutation. For example, the FDA has approved a combination of dabrafenib and trametinib for the treatment of anaplastic thyroid cancer patients, carrying the BRAF^Val600Glu^ mutation (41).

*In silico* modeling of the BRAF proteins was performed using the Swissmodel and Hope tools. It was observed that the overall structure of the protein (**Supplementary Figure 1**) was not altered (QMEAN score 0.57 ± 0.05 for both models); however, the mutation causes a change in residue Val600Glu. The mutated residue is located in a domain that is crucial for the activity of the protein, thus the mutation affects its function and interaction with other proteins.

A small number of studies have researched the role of KIT in PTC. Sanlorenzo et al. (42) mentioned that the proto-oncogene KIT encodes for the tyrosine kinase receptor and is involved in cell signal transduction with different downstream pathways: MAPK, phosphatidylinositol 3-kinases (PI3K), Janus kinase (JAK)/signal transducers and activators of transcription (STAT), SRC family kinases (SFK) and phospholipase Cγ (42). On the other hand, the mitogen-activated protein kinase (MAPK) pathway has been extensively researched, and the role of point mutations in the BRAF and RAS genes and RET/PTC rearrangements in PTC molecular pathogenesis has been described (43, 44).

*In silico* modeling of the KIT proteins was analyzed using the Swiss-Model and Hope tools. It was observed that the overall structure of the protein (**Supplementary Figure 2**) was not altered (QMEAN score 0.71 ± 0.05 for both models), but the mutation causes a change in residue Leu678Phe. The mutated residue is located in a domain that is important for binding of other molecules and also is in contact with residues in a domain that is important for the activity of the protein. Therefore, the mutation might disturb the interaction between protein domains and as such affect the function of the protein.

The 3D molecular structure obtained using the HOPE tool shows that the mutant residue is bigger than the wild-type residue (**Supplementary Figures 1**, **2**). The wild-type residue was buried in the core of the protein, and the fact that it is bigger, probably causes that it does not fit.

The precise role of KIT in cancer is still unknown, and numerous investigations present discrepancies depending on the type of tumor. Studies show that it is highly expressed or mutated in small cell lung cancer, leukemia cells, colon cancer, and neuroblastoma. Other studies demonstrate that the KIT expression is lost in breast cancer and melanoma (8). Similarly, a low expression of the KIT gene has been reported during the transformation of normal thyroid epithelium to papillary carcinoma, suggesting a possible role of the gene in the differentiation of thyroid tissue (8, 45, 46).

The strength of the used approach relies on the high analytical sensitivity of NGS. NGS can efficiently detect variants even in tissue samples; moreover, it allows to analyze several genes at once, compared to Sanger (∼1000 bp at once) (47). On the other hand, NGS limitations include higher costs and the need of specialized bioinformatic tools (48).

To conclude, we believe that this report represents the genetic characteristics of PTC in an Ecuadorian woman with a mainly Native American ethnic component. Further studies of pathological variants are needed to determine if the combined demographic and molecular profiles are useful to develop targeted treatments focused on the Ecuadorian population.

## Data availability statement

The original contributions presented in the study are included in the article/**Supplementary Material**. Further inquiries can be directed to the corresponding author.

## Ethics statement

The studies involving human participants were reviewed and approved by CEISH- UTE (CEISH-2021-014). The patients/participants provided their written informed consent to participate in this study. Written informed consent was obtained from the patient for publication of this case report.

## Author contributions

Conceptualization, SC-U, EP-C, RT-T and AZ. Resources, PS-P, CG, RG, EL-N, AZ. Methodology, AZ, SC-U, EP-C, RT-T, PG-R and VR-P. Formal Analysis, SC-U, EP-C, RT-T and AZ. Writing – Review and Editing, SC-U, EP-C, RT-T and AZ, PS-P. Supervision, AZ. Project Administration, AZ. Funding Acquisition, AZ. All authors contributed to the article and approved the submitted version.

## Supplementary material

Supplementary Figure 1 Three-dimensional structures of wild and mutant proteins, obtained with the Swissmodel tool. **(A)** Wild Type BRAF Protein (P15056, extracted from UniProt). **(B)** Mutant protein (p.Val600Glu). **(C)** Close-up of the BRAF genetic mutation identified in the patient by NGS. The wild-type residue is in red, whereas the mutant residue is in green.

Three-dimensional structures of wild and mutant proteins, obtained with the Swissmodel tool. **(A)** Wild Type KIT Protein (P10721-1, extracted from UniProt). **(B)** Mutant protein (p.Leu678Phe). **(C)** Close-up of the mutation. The KIT protein is colored grey, the side chains of both, the wild-type and the KIT^Leu678Phe^ variant residue are shown and colored green and red respectively.
