# Supplementary figures and images for: Identification of KIT and BRAF mutations in thyroid tissue using next-generation sequencing in an Ecuadorian patient: A case report
Source: Front Oncol. 2023 Jan 17;12:1101530. doi: 10.3389/fonc.2022.1101530 (PMC9887188; doi:10.3389/fonc.2022.1101530)

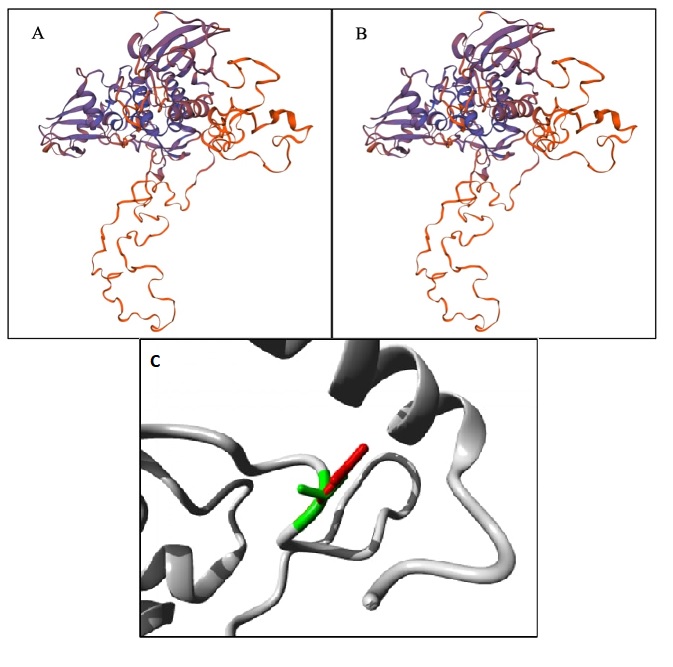

Supplement: Supplementary Figure 1 — Three-dimensional structures of wild and mutant proteins, obtained with the Swissmodel tool. (A) Wild Type BRAF Protein (P15056, extracted from UniProt). (B) Mutant protein (p.Val600Glu). (C) Close-up of the BRAF genetic mutation identified in the patient by NGS. The wild-type residue is in red, whereas the mutant residue is in green. [file Image_1.jpg]

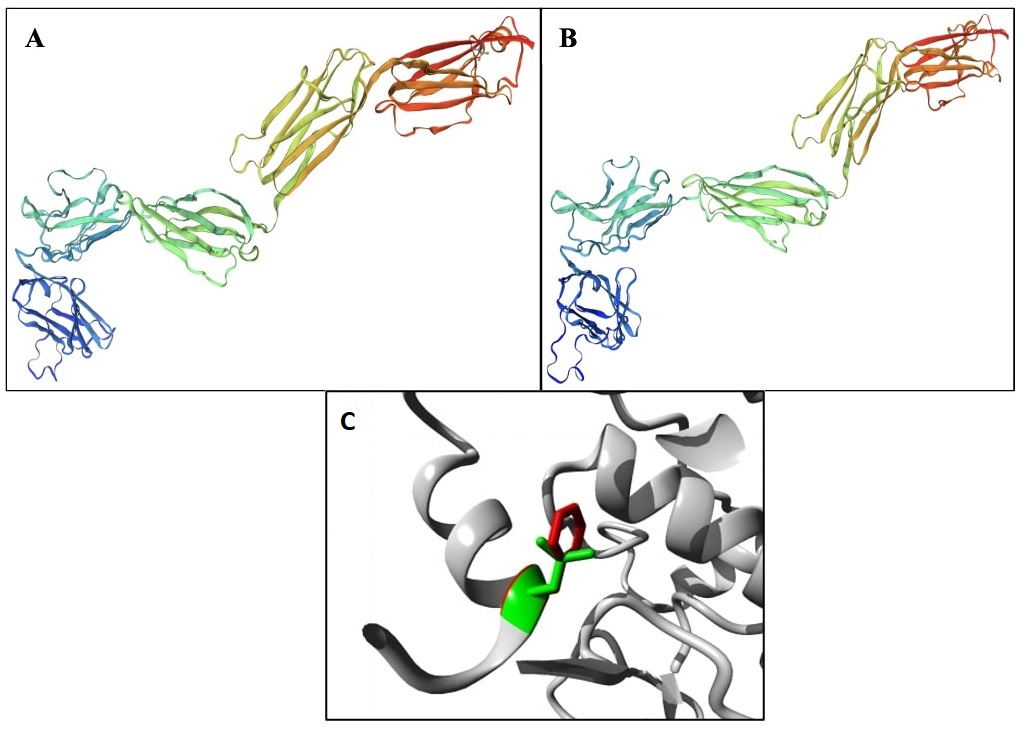

Supplement: Supplementary file 2 — Three-dimensional structures of wild and mutant proteins, obtained with the Swissmodel tool. (A) Wild Type KIT Protein (P10721-1, extracted from UniProt). (B) Mutant protein (p.Leu678Phe). (C) Close-up of the mutation. The KIT protein is colored grey, the side chains of both, the wild-type and the KITLeu678Phe variant residue are shown and colored green and red respectively. [file Image_2.jpg]
